# Clinical Recommendations for Implant Verification Jigs in Analogue and Digital Workflows. A Narrative Review

**DOI:** 10.1111/adj.70025

**Published:** 2025-11-25

**Authors:** Aaron Wai Harng Wong, Robert Nedelcu, Adam Hamilton

**Affiliations:** ^1^ University of Western Australia Crawley Western Australia Australia

**Keywords:** implant superstructure, implant verification jigs, misfit

## Abstract

**Background:**

Misfit at the implant–prosthesis interface arises from small errors that occur during impression‐taking, cast fabrication and milling. Implant verification jigs (IVJs) have been described as a quality‐control device in fixed implant prosthodontics, yet protocols and indications are inconsistently described This narrative review aims to compare materials and fabrication methods, map their functional roles and provide clinical recommendations for both analogue and digital workflows.

**Methods:**

Peer‐reviewed English‐language publications from 1 January 1980 to 30 October 2025 were searched on PubMed and Scopus using MeSH and free‐text terms for dental implants and IVJs. Eligible studies include technique reports, in vitro studies, clinical studies and narrative reviews that reported materials, fabrication or functional roles of IVJs in fixed implant prosthodontics. Titles, abstracts and full texts were screened against predefined criteria. Data were synthesised narratively by workflow and verification approach.

**Results:**

Fifty‐two publications met the criteria (29 technique descriptions, 12 in vitro, 8 in vivo, 1 case report, 2 reviews). The materials reported for IVJs included PMMA, UDMA, photopolymerising composite resins and Type III/IV dental stone jigs. In analogue workflows, intra‐oral and extra‐oral verification procedures have been reported. In digital workflows, digitising IVJs or implant verification casts as well as CAD‐CAM IVJs have been described.

**Conclusions:**

Within the limits of this narrative review, IVJs can be used as adjuncts for conventional impressions and intra‐oral scans with vertical scanbodies. Photogrammetry and systems that pair horizontal scanbodies and AI‐based recognition provide datasets with greater accuracy, negating the need for conventional IVJs. Future studies should standardise verification strategies and evaluate long‐term clinical outcomes.

**Clinical Relevance:**

This narrative review clarifies the indications for IVJs, outlines workable analogue and digital strategies, and provides clinical recommendations. Selective use of IVJs prior to the fabrication of the definitive prosthesis can reduce the likelihood of misfit.

## 1 Introduction

Passive fit denotes a stress‐free assembly whereby the implant prosthesis seats with simultaneous even contact producing no strain in the dental implants, prosthetic components and peri‐implant bone [1]. Jemt [2] described passive fit more broadly as a level of fit that does not lead to long‐term complications, with misfit magnitudes < 150 μm cited as acceptable. In practice, an absolute fit with zero strain is impossible to achieve as inaccuracies are introduced during impression taking, [3, 4, 5, 6, 7, 8, 9, 10] cast fabrication [11, 12] and manufacturing of the prosthesis [9, 13]. Although errors at each step do not contribute equally, or in a simple additive manner to overall misfit, larger operator‐ and laboratory‐introduced deviations, combined with normal industrial manufacturing variability, can still yield a greater clinical misfit. Hence, risk mitigation should focus on driving misfit below levels likely to precipitate biological or mechanical complications [1]. However, a universal numerical threshold for clinically acceptable misfit has not been established due to methodological heterogeneity and inconsistent clinical correlations between misfit and complications [14, 15].

Digital workflows shift where errors arise and how they are detected. Although intra‐oral scanning has shown comparable in vitro accuracy, [16, 17, 18] clinical conditions, such as saliva, limited access and patient movement can magnify discrepancies [19]. As an alternative, photogrammetry has demonstrated greater precision and trueness than intra‐oral scanners by mitigating soft‐tissue interferences and stitching artefacts [20, 21, 22, 23, 24]. Emerging systems that pair horizontal scan bodies with AI‐based recognition also report superior trueness and precision, although the published studies remain scarce [25, 26, 27, 28]. However, these approaches require dedicated scanning flags and remain dependent on meticulous component seating.

In vivo research on various techniques is limited. However, the trueness of models manufactured from non‐splinted impressions (28 ± 7 μm) and modified intra‐oral scan (IOS) acquisition (41 ± 11 μm) of six maxillary implants using a modified scan procedure showed no statistically significant difference [9]. On the other hand, manufactured implant‐supported frameworks posed a statistically significant difference in misfit to both impression‐based models and IOS acquisition with a trueness of (70 ± 23 μm).

Unlike analogue workflows, printed resin master casts are typically outside the manufacturing pathway, with the dimensional variability reflecting printing and post‐processing and not verifying the intra‐oral implant positions [29, 30]. Full‐contour prototypes can be utilised in edentulous patients to verify fit, aesthetics and occlusion, but the material compliance and soft‐tissue contact can mask discrepancies [28, 31]. In partially dentate patients, proximal contact with the remaining dentition can also compromise the verification process, further reinforcing the need for an explicit verification step.

Implant verification jigs (IVJs) have been described as a quality‐control device with the potential to confirm that the intra‐oral implant positions have been accurately transferred to the master cast before prosthesis fabrication [32, 33]. Their proposed role is to detect discrepancies at the implant–prosthetic interface and enable corrective measures to reduce the risk of clinically detectable misfit at the implant–abutment interface [34, 35]. Unlike splinted open‐tray impressions, which aim to reduce error during impression‐taking, IVJs are used to evaluate the outcome of impression‐taking and cast fabrication. Removal of the soft‐tissue mask and inspecting the connection under magnification have been suggested as means to assess the implant–prosthetic interface on cast models without confounders. While an IVJ cannot guarantee passive fit, it may reduce the risk of misfit by intercepting errors prior to the fabrication of the definitive prosthesis.

With passive fit unattainable due to small stepwise errors accumulating across analogue and digital workflows, IVJs provide an additional verification step before prosthesis fabrication. However, the literature is highly heterogeneous and fragmented, with protocols poorly defined and indications unclear.

This narrative review aims to compare materials and fabrication methods, map their functional roles, and provide clinical recommendations based on the available scientific evidence to support the use of IVJs in both analogue and digital workflows.

## 2 Materials and Methods

Peer‐reviewed English‐language publications from 1 January 1980 to 30 October 2025 were searched on PubMed and Scopus using MeSH and free‐text keywords for dental implants and IVJs. Boolean strings were adapted to each database and listed in Table 1. Reference lists and citing articles were hand‐searched to identify additional articles.

**TABLE 1:** Search strategy using PubMed regarding ‘implant verification jigs in implant dentistry’.

Database	Boolean string	Results
PubMed	(verification) AND (dental implant)	195
Scopus	TITLE‐ABS‐KEY((verification) AND (dental implant*))	209

References were imported into Endnote 21 (Clarivate, Philadelphia, PA), and duplicates were identified and removed. Titles and abstracts were screened for relevance to implant verification, followed by full‐text analyses. Eligible studies include technical descriptions, case reports, in vitro studies, clinical studies and narrative reviews that reported on materials, fabrication methods and functional roles of IVJs in fixed implant prosthodontics. Reasons for exclusion were documented. From each included paper, the study design, workflow type, comparisons or outcome measures and functional roles were extracted. Findings were synthesised narratively and the evidence grouped by analogue and digital workflows, followed by materials, fabrication methods and functional roles.

## 3 Results

### 3.1 Study Selection

Database search identified 404 publications using the search strategy. One hundred and fifty‐eight duplicates were removed, leaving 246 unique publications. Screening of the titles and abstracts excluded 174 irrelevant publications. Hand‐searching included seven additional publications. Full text analysis was conducted on 65 publications from which 52 publications were included that were made up of 29 technique descriptions, 12 in vitro studies, 8 in vivo studies, 1 case report and 2 reviews.

### 3.2 Study Characteristics and Synthesis

The study design, workflow type, comparisons or outcome measures, and functional roles for both analogue and digital workflows are presented in Tables 2, 3, 4. Analogue workflows dominated the earlier literature, whereas more recent publications described digital adaptations of the verification process.

**TABLE 2:** Summary of peer‐reviewed publications on analogue workflows using IVJs, classified by study design, materials and functional roles.

Author and Year	Study design	Materials	Functional roles
Knudson, Williams et al. 1989 [32]	Technique description	Cyanoacrylate and orthodontic wire	Verification (intra‐oral)
Papazian and Morgano 1998 [36]	Technique description	Cyanoacrylate and aluminium strips	Verification (extra‐oral)
Blasi, Henarejos‐Domingo et al. 2022 [37]	Technique description	Dental stone	Verification (intra‐oral) Correction (new impression)
Arias, Londono et al. 2022 [38]	Technique description	PMMA	Verification
Balshi and Wolfinger 1996 [39]	Technique description	PMMA and denture	Verification (extra‐oral) Interocclusal records
Hebel, Galindo et al. 2000 [35]	Technique description	PMMA	Verification (extra‐oral) Correction (implant verification cast)
Henry 1987 [40]	Technique description	PMMA and orthodontic wire	Verification (extra‐oral) Correction (repositioned implant replica) Interocclusal records
Maalhagh‐Fard 2002 [41]	Technique description	PMMA	Verification (intra‐oral) Correction (new impression) Interocclusal records
McCartney 1991 [42]	Technique description	PMMA and metal framework	Verification (extra‐oral) Correction (repositioned implant replica)
McCartney and Doud 1993 [43]	Technique description	PMMA and metal framework	Verification (extra‐oral) Correction (repositioned implant replica)
McCartney and Pearson 1994 [33]	Technique description	PMMA	Verification (extra‐oral) Correction (repositioned implant replica)
Rasmussen 1987 [44]	Technique description	PMMA	Verification (intra‐oral) Correction (new impression)
Vitale, Tung et al. 2009 [45]	Technique description	PMMA	Verification (intra‐oral) Correction (repositioned implant replica)
Suh, Park et al. 2025 [46]	Technique description	PMMA and surgical guide	Correction (implant verification cast)
Figueras‐Alvarez and Real‐Voltas 2021 [47]	Technique description	PMMA, dental stone or wax	Verification (extra‐oral)
Al‐Abbas, Al‐Ajmi et al. 2002 [48]	Technique description	UDMA	Verification (intra‐oral)
Salloum 2025 [49]	Technique description	UDMA	Verification (extra‐oral) Correction (new impression)
Silverstein, Kurtzman et al. 2002 [50]	Technique description	UDMA	Verification (extra‐oral)
Su, Tsai et al. 2021 [51]	Technique description	Methacrylate‐based photopolymerised resin with photopolymerising composite resin	Verification (extra‐oral) Correction (repositioned implant replica, implant verification cast)
Papaspyridakos, Rajput et al. 2017 [52]	Case report	PMMA and denture	Verification (extra‐oral) Interocclusal records
Alhashim and Flinton 2014 [53]	Case report	Dental stone	Verification (intra‐oral)
Goll 1991 [54]	Literature review	PMMA and dental bur or floss Cyanoacrylate or composite resin and orthodontic wire	Verification (intra‐oral) Correction (implant verification cast)

**TABLE 3:** Summary of peer‐reviewed publications on digital workflows using IVJs, classified by study design, materials/technique, and functional roles.

Author and year	Study design	Material/technique	Functional roles
Lin, Harris et al. 2014 [55]	Technique description	PMMA	Verification (extra‐oral) Correction (repositioned implant replica)
Negreiros, Chanting Sun et al. 2023 [56]	Technique description	PMMA	Verification (extra‐oral) Correction (digitised implant verification cast)
Rosmaninho, Vedovato et al. 2024 [57]	Technique description	PMMA	Verification (extra‐oral) Correction (digitised IVJ with reverse scan bodies)
Rosmaninho, Vedovato et al. 2025 [58]	Technique description	PMMA	Verification (extra‐oral) Correction (digitised IVJ with reverse scan bodies)
Sinada & Papaspyridakos 2021 [59]	Technique description	Milled PMMA prosthesis	Verification (intra‐oral)
Guichet and Paquette 2025 [60]	Technique description	Milled PMMA prosthesis	Verification (intra‐oral) Interocclusal records
Hyspler, Strnad et al. 2025 [61]	Technique description	PMMA and milled PMMA prosthesis	Verification (extra‐oral) Correction (digitised IVJ with reverse scan bodies) Interocclusal records
Liaropoulou, Kamposiora et al. 2024 [62]	Technique description	PMMA and milled PMMA prosthesis	Verification (extra‐oral) Correction (digitised IVJ with reverse scan bodies) Interocclusal records
Altoman, Alshowail et al. 2025 [63]	Technique description	Photogrammetry Milled PMMA prosthesis	Verification (extra‐oral & intra‐oral) Correction (photogrammetry) Interocclusal records
Betita & Rusthoven 2025 [64]	Literature review	Photogrammetry	Verification (extra‐oral) Correction (photogrammetry)

**TABLE 4:** Summary of peer‐reviewed in vitro and in vivo publications on analogue and digital workflows using IVJs, classified by study design, workflow type, outcomes and functional roles.

Author and year	Study design	Workflow	Material/technique	Comparison/outcome measures	Functional roles
Manzella, Bignardi et al. 2016 [65]	In vitro	Analogue	Dental stone	Can detect 50 μm vertical misfit, 150 μm horizontal misfit, 1^o^ angular discrepancy	Verification (simulated intra‐oral)
Aljohani, Bukhari et al. 2022 [66]	In vitro comparative	Analogue	PMMA Dental stone Composite resin	PMMA ≈ dental stone Composite resin with significantly greater distortions	Correction (implant verification cast compared with research reference)
Basaki, Alkumru et al. 2017 [67]	In vitro comparative	Analogue	PMMA	3D implant deviation of milled casts (116 ± 94 μm) was significantly greater than conventional casts (56 ± 29 μm) Passivity of conventional (18/20) was significantly greater than milled casts (11/20)	Correction (implant verification cast used as research reference)
Carr and Master 1996 [68]	In vitro comparative	Analogue	PMMA and metal framework	Distortion of implant verification cast (57 μm) similar to splinted PVS impressions (41 μm)	Verification (simulated extra‐oral) Correction (implant verification cast)
De La Cruz, Funkenbusch et al. 2002 [69]	In vitro comparative	Analogue	PMMA UDMA	IVJs not significantly more accurate than impressions	Correction (implant verification cast compared with reference cast)
Kurella, Nesappan et al. 2020 [70]	In vitro comparative	Analogue	PMMA	No difference between high‐rigid vinyl polyxiloxane, polyvinyl siloxane and polyether impressions No difference between splinted and non‐splinted impressions	Correction (implant verification cast compared with reference cast)
Masu, Tanaka et al. 2021 [71]	In vitro comparative	Analogue	PMMA and cobalt–chromium framework	Precision of intra‐oral scans with assistive devices using Primescan and True Definition scanner was significantly better than implant verification casts	Verification (simulated intra‐oral) Correction (implant verification cast)
Papaspyridakos, Kim et al. 2017 [72]	In vitro comparative	Analogue	PMMA UDMA	PMMA ≈ UDMA	Correction (implant verification cast compared with reference cast)
Manzella, Burello et al. 2013 [73]	In vivo	Analogue	Dental stone	55% of IVJs broke No breakages with second IVJ after correction	Verification (intra‐oral) Correction (repositioned analogues)
Ashida, Sanda et al. 2025 [74]	In vivo comparative	Analogue	PMMA and cobalt–chromium framework	Precision of intra‐oral scans with assistive devices was superior to intra‐oral scans without assistive devices and IVJs	Correction (implant verification cast used as research reference)
Chochlidakis, Papaspyridakos et al. 2020 [75]	In vivo comparative	Analogue	PMMA	3D implant deviations between digital and verified conventional casts were 162 ± 77 μm	Verification (extra‐oral) Correction (implant verification cast used as research reference)
Ercoli, Geminiani et al. 2012 [34]	In vivo comparative	Analogue	PMMA and dental floss or orthodontic wire	IVJ significantly improved passive fit of full‐arch implant‐supported fixed dental prosthesis	Verification (extra‐oral) Correction (implant verification cast)
Gedrimiene, Adaskevicius et al. 2019 [76]	In vivo comparative	Analogue	NR	Significant differences between intra‐oral scans and verified conventional casts for distance between scan bodies (70.8 ± 59 μm), rotation (2.0 ± 1.37^o^) and vertical shift (82.2 ± 61.7 μm)	Verification (extra‐oral) Correction (implant verification cast used as research reference)
Papaspyridakos, Benic et al. 2012 [77]	In vivo comparative	Analogue	UDMA	Splinted impressions produced more accurate casts than non‐splinted impressions	Verification (extra‐oral) Correction (implant verification cast as research reference)
Papaspyridakos, De Souza et al. 2023 [78]	In vivo comparative	Analogue	NR	3D implant deviations between digital and verified conventional casts were 88 ± 24 μm No differences between maxillary and mandibular arches	Verification (extra‐oral)
Papaspyridakos, Lal et al. 2011 [5]	In vivo comparative	Analogue	PMMA	Prostheses from splinted impressions more accurate than non‐splinted impressions All prostheses from implant verification casts fit	Verification (extra‐oral) Correction (implant verification cast)
Nuytens, D'Haese et al. 2022 [79]	In vivo comparative	Analogue	PMMA	Passive fit noted with analogue IVJs Digital bite registration using adjustable bite pillars was faster but significantly deviated from analogue bite registration	Verification (extra‐oral) Interocclusal records (wax rim)
Roig, Roig Cayón et al. 2022 [80]	In vivo comparative	Analogue	Dental plaster	Intra‐oral scans with auxiliary device allow monolithic zirconia prostheses with better fit than verified conventional casts	Verification (intra‐oral)
Nagai, Kuroda et al. 2025 [81]	In vitro comparative	Digital	Milled PMMA prosthesis luted with titanium cylinders	No statistical difference between scanning protocols (splinted scan bodies vs. reverse scan bodies) and prosthesis type (FP‐1 vs. FP‐3)	Verification (simulated intra‐oral)
Asavanant, Yang et al. 2025 [82]	In vitro comparative	Digital	PMMA (milled vs. 3D printed)	Milled IVJ with less misfit than 3D printed IVJ	Verification (simulated intra‐oral)

Within analogue workflows, polymethylmethacrylate (PMMA) and Type IV dental stone were commonly used. PMMA IVJs were typically sectioned and reconnected to limit polymerisation shrinkage. Type IV dental stone IVJs act as a rigid index that will fracture when excessive discrepancy at the implant–abutment interface is present, providing an objective measure of misfit. Comparative in vitro studies found no significant differences between PMMA, photopolymerising urethane dimethacrylate (UDMA) and dental stone, whereas photopolymerising composite resin exhibited significantly greater distortions [66, 72]. Three functional roles were described. First, verification of the transfer of intra‐oral implant positions to the master cast is achieved by extra‐oral fabrication with intra‐oral assessment, or intra‐oral fabrication with extra‐oral assessment. Second, correction is achieved by using the IVJ to retake splinted open‐tray impressions, fabricate an implant verification cast (IVC), or guide repositioning of implant replicas on the master cast. Lastly, maxillomandibular relationships may occasionally be recorded. Evidence on the downstream clinical benefit is conflicting, with some showing no advantages, [68, 69] whereas other studies found a higher likelihood of passive fit when IVJs are used [5, 34, 73].

Publications on digital workflows were mainly technique reports, with three approaches described. First, a hybrid approach involving an analogue IVJ or IVC can be used to verify or correct IOSs or printed resin master casts. Secondly, photogrammetry captures implant positions using dedicated scanning flags and specific scanners to verify and correct IOSs. Lastly, digitally designed PMMA IVJs can be milled or 3D printed for intra‐oral verification and interocclusal records. Across these publications, outcomes mainly demonstrate clinical feasibility, with few clinical end‐points reported.

Most studies reported dimensional accuracy of master casts or digital datasets, with simple assessments of fit with the Sheffield one screw test or best‐fit alignment. Few studies used the fracture of dental stone IVJs as a binary end‐point for excessive misfit. Clinical end‐points were scarce.

## 4 Discussion

Verification of conventional implant impressions is well described in the literature, with recent case reports and technique articles outlining adaptations for digital workflows. However, the published techniques are heterogeneous in how verification is performed and integrated into prosthodontic workflows, and they often rely on subjective assessment parameters to determine clinical acceptability. In the following discussion on the literature identified by this narrative review, the commonly reported techniques and workflows will be disseminated and their limitations highlighted.

### 4.1 Analogue Workflows—Materials and Functional Roles

Within analogue workflows, material choice follows function. An IVJ should be rigid, dimensionally stable, free of soft‐tissue contact, easy to fabricate and modify, resistant to handling deformation, and ideally provide an objective cue of misfit. PMMA and UDMA IVJs are practical and can improve the passive fit of full‐arch implant‐supported prostheses, yet they remain dependent on subjective fit assessments [34]. Dental stone IVJs are harder to fabricate and modify, but they can provide a simple fracture end‐point when gross misfit is present [37, 53, 65, 73, 80]. Photopolymerising composite resins tend to distort, [51, 66] whereas cyanoacrylate is only suitable to stabilise segments; thus they are best avoided [32, 36, 54]. No single material meets all ideal requirements. When PMMA, UDMA and Type IV dental stone are handled correctly, the performance is broadly comparable, so the fabrication technique and how fit is assessed play a bigger role than the material choice [66, 69, 72].

**FIGURE 1:**
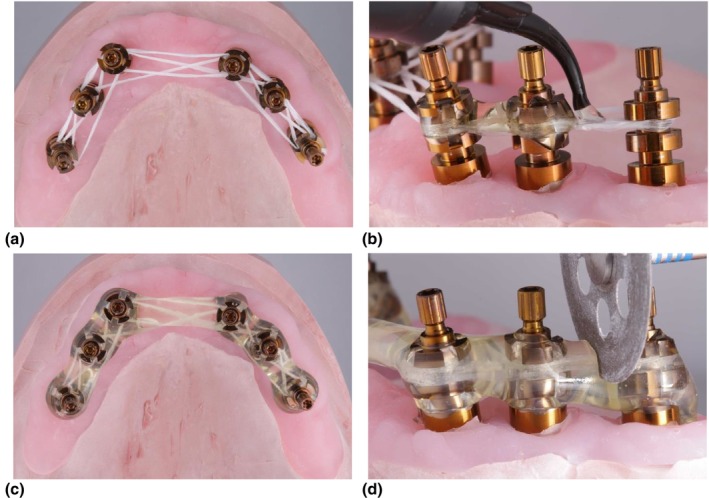
PMMA IVJs fabricated with (a) floss to support the creation of a (b–c) rigid resin framework which is (d) sectioned with a disc and rejoined to release any polymerisation stress.

**FIGURE 2:**
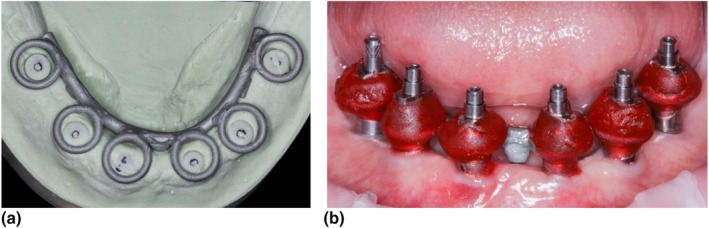
(a) a rigid cast metal base metal framework is fabricated based on a preliminary impression and then (b) joined to non‐engaging impression copings or provisional cylinders intra‐orally with self‐curing acrylic resin.

**FIGURE 3:**
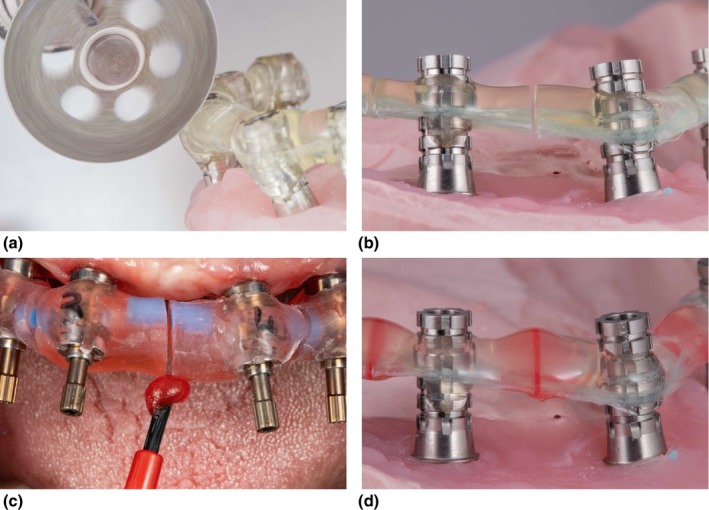
(a–b) IVJs thinly sectioned extra‐orally with a diamond disc, (c) reconnected intra‐orally with the Nealon brush technique and (d) seated on the master cast to verify the implant positions prior to fabrication of the definitive prosthesis.

PMMA is a pragmatic choice for IVJs for partially dentate and fully edentulous patients [5, 33, 35, 38, 41, 44, 45, 47, 66, 67, 70, 75, 79]. Accuracy depends on controlling material bulk and polymerisation shrinkage when rigidly splinting non‐engaging provisional abutments or impression copings on multi‐unit abutments or implants with external connections [83]. Adjuncts such as floss (Figure 1), dental burs, removable prostheses, surgical guides and rigid metal frameworks (Figure 2) can guide resin placement, [34, 39, 42, 43, 46, 52, 54, 68, 71, 74] whereas heavy gauge orthodontic wires are contraindicated as their elastic memory and stiffness can distort the IVJ [32, 40, 84]. After initial polymerisation, thinly sectioning followed by minimally re‐luting the splint with the Nealon brush technique minimises distortion caused by polymerisation shrinkage while maintaining cohesion (Figure 3) [33, 35, 45, 85, 86, 87]. However, some microstrain inevitably remains within the rigid construct, highlighting the inherent limitations of PMMA as a splinting material [88].

Low‐expansion Type III and Type IV dental stone IVJ are acceptable alternatives that provide stiff, dimensionally stable indices with a simple fracture end‐point when gross misfit is present [37, 53, 65, 66, 73]. Some drawbacks include a technically more demanding fabrication and modification process, with intra‐oral reconnection of segments using dental plaster [37, 73]. Photopolymerising UDMA resin IVJ, despite having higher levels of polymerisation shrinkage, can perform comparably to PMMA IVJs when the same section‐and‐reconnect principles are applied [48, 49, 50, 69, 72, 77, 89].

**FIGURE 4:**
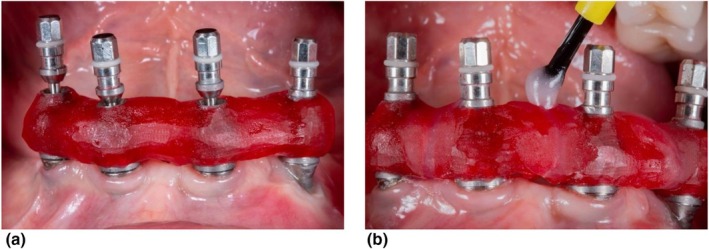
(a) Assessment of a verification jig fit using visual inspection and the Sheffield One Screw Test showing misfit on the mandibular right posterior and anterior implants when the screw is tightened in the left most posterior implant. (b) Rejoining following sectioning of the verification jig and again performing the Sheffield One Screw Test with no misfit detected.

Verification is performed after impression‐taking and cast fabrication to confirm that the implant positions have been faithfully replicated. For resin IVJs, two pathways are available. The IVJ can be fabricated on the cast and allowed a 24‐h setting period to ensure dimensional stability, followed by an intra‐oral assessment of fit using methods such as rocking, tactile sensation, visual inspection and Sheffield One screw test (Figure 4) [41, 44, 45, 48, 54, 75, 90]. However, soft‐tissue interference, saliva, screw preload and limited access with subgingival and internal connections can mask misfits [91, 92, 93]. Alternatively, they can be fabricated and sectioned on the cast, followed by intra‐oral reconnection, allowing a recommended 17 min for initial polymerisation of low shrinkage self‐cure acrylic resin before removal [5, 33, 34, 35, 36, 37, 39, 40, 42, 43, 47, 49, 50, 51, 52, 68, 73, 76, 77, 78, 79, 90]. This pathway is preferred as the implant–abutment interface can be assessed on the master cast under magnification with the soft‐tissue mask removed [92]. As no single modality is definitive, clinicians must combine these evaluations with clinical judgement to determine the need for corrective measures [91, 92, 93]. Contrarily, dental stone IVJs are fabricated extra‐orally and assessed intra‐orally, with the fracture on seating providing an objective cue for gross misfit and prompting the need for correction; however, the threshold for fracture depends on the thickness, inter‐implant distances and properties of the stone which can lead to highly variable fracture thresholds [37, 53, 65, 73, 80].

**FIGURE 5:**
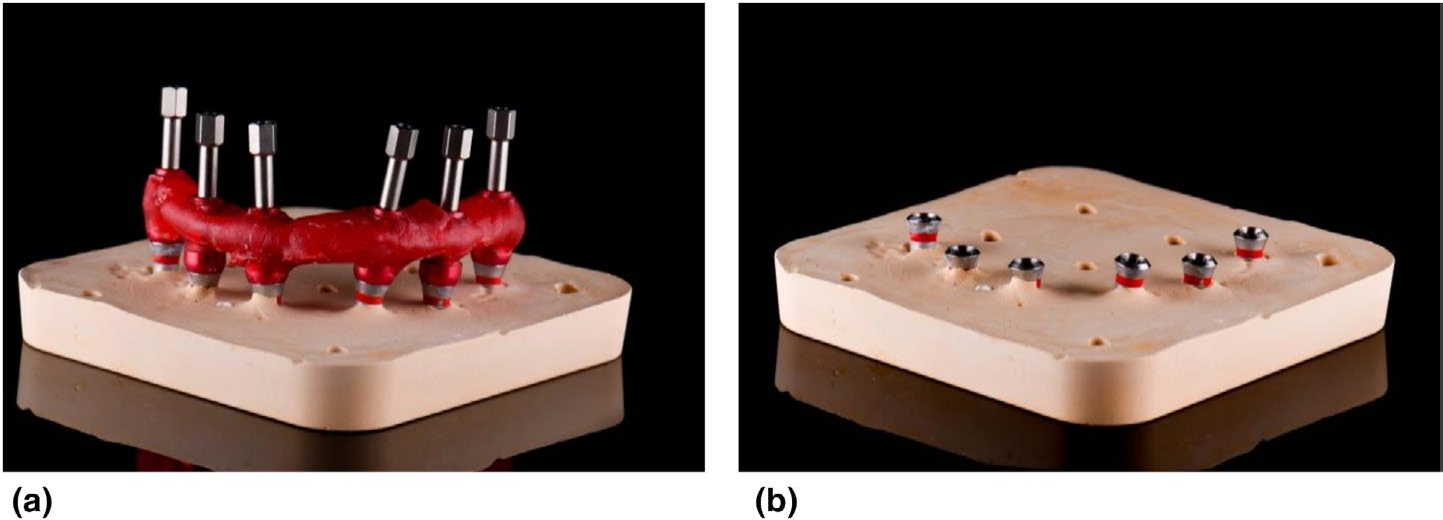
(a) IVJ made with GC pattern resin splinting multiple impression copings used to fabricate an (b) implant position cast.

Correction of the master cast is the secondary function that is guided by the seating pattern of the IVJ. A localised discrepancy with stable seating elsewhere indicates a malpositioned implant replica on the master cast. However, identification of the individual implant that has a discrepancy can be challenging and can be challenging if multiple misfits are present. Correction can be confined to that site by removing and repositioning the implant replica on the master cast while using the IVJ as the spatial reference [33, 40, 42, 43, 45, 51, 73]. Soft‐tissue architecture can be replicated using a polyether impression taken prior to correction [45]. A generalised mismatch suggests a gross error with impression‐taking or cast fabrication; thus, the IVJ may be used to retake a splinted open‐tray impression [33, 37, 41, 44, 49] or fabricate a separate IVC [5, 34, 35, 46, 51, 54, 66, 68, 69, 70, 71, 72] (Figure 5). Although this generates a new dataset independent of the original master cast that should be verified accordingly, it is frequently adopted as the reference comparator in clinical studies, signalling clinician confidence in their accuracy [67, 74, 75, 76, 77].

In edentulous arches with no stable landmarks, an IVJ can provide a stable scaffold for interocclusal records. This is best captured after the master cast has been verified to avoid embedding errors. An elastomeric interocclusal recording material or autopolymerising acrylic resin may be used, with hard wax or conversion prostheses acting as a carrier when required [39, 40, 41, 52]. Wax rims should be avoided due to the risk of distortion [79]. In this context, the IVJ functions as a passive carrier rather than a record base.

When the accuracy of a conventional impression is in question, IVJs can be used to verify the accuracy of master casts. A thinly sectioned and intra‐orally reconnected PMMA or UDMA IVJ assessed on the master cast confirms that the implant positions have been faithfully replicated, while dental stone IVJs can provide objective fracture cues for gross misfit when used intra‐orally. If verification is unsatisfactory, a localised correction of the master cast using the IVJ as a spatial reference, splinted open‐tray impressions or IVCs can minimise the misfit prior to fabrication of the definitive prosthesis and avoid the potential need to remake the prosthesis. Where the fabrication costs of a framework are not a barrier, the accuracy of the impression and workflow can be assessed following fabrication at the framework try‐in stage without prior verification. However, in the event of a clinically unacceptable prosthesis misfit where often a complete remake of the framework is required, the question will emerge—will repeating the same original impression technique lead to a better outcome?

### 4.2 Digital Workflows—Verification Challenges and Strategies

**FIGURE 6:**
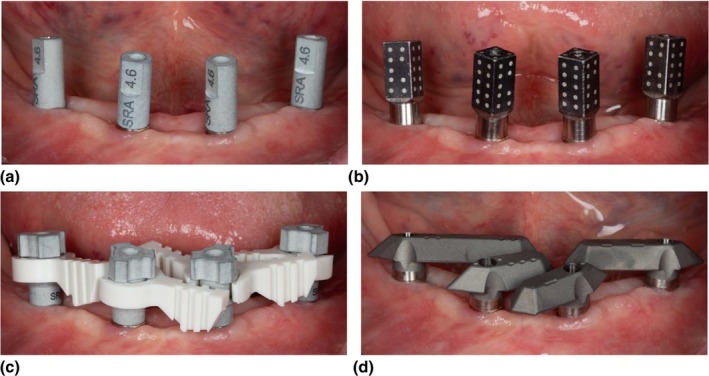
Scanning flags seated on multi‐unit abutments to capture implant positions with (a) IOS and vertical scan bodies which are most affected by soft‐tissue stitching errors, (b) stereophotogrammetry with an extra‐oral scanner, (c) IOS vertical scan bodies connected with a scan ladder to reduce soft‐tissue influence, and (d) IOS using horizontal scan body system with post‐scanning AI corrections and repeated scan validation.

Digital workflows have had significant advantages in implant scanning with more efficient workflows; however, since the data are in a digital format and a model or cast is no longer a sequential part of the workflow which leads to the fabrication of the prostheses, this changes the target of verification. Given the variation in precision reported with IOS and vertical scan body systems (Figure 6a) due to significant patient and operator factors, specifically its reliance on soft tissue which can impact its accuracy, [94, 95, 96] it would suggest that verification would be prudent particularly in situations of long‐spans between implants and lack of fixed keratinised mucosa. Furthermore, many prostheses fabricated from digital scans are monolithic full‐contour restorations in which there is no opportunity to perform a traditional framework try‐in procedure where there is no soft‐tissue contact, or in partially edentulous situations proximal contacts, which will significantly affect the ability to clinically evaluate the fit of the prosthesis using tactile sensation or the Sheffield one screw test.

Due to considerable variation in 3D printing accuracy, printed resin master casts cannot be considered a reflection of the trueness of the IOS. Printed models also do not form part of the sequential workflow in the manufacturing of the prosthesis, as a CAD/CAM framework is made directly from the original scan data. Techniques for verifying and correcting a printed master cast as reported in the literature have limitations with additional errors introduced when the printed model is rescanned [29, 30, 55, 75, 76, 78]. Alternate techniques have been proposed in which a milled PMMA IVJ that is digitally designed from the IOS can be seated intra‐orally to gauge passivity, while capturing interocclusal records in edentulous patients [59, 60, 63]. This approach can be further modified to include the use of reverse scan bodies for digitisation [61, 62]. However, it has greater limitations compared with analogue IVJs, with the inherent flexibility of the PMMA prototype used as the IVJ with the connections having no metal interfaces and being in full contour with soft‐tissue contact making assessment of fit at the implant–prosthetic interface unreliable. The intra‐oral fit assessment is subjective; soft‐tissue contact and screw preload can mask discrepancies, and radiolucency limits the appraisal of subgingival interfaces. Material compliance may also permit distortion and apparent seating under preload. There is also no straightforward pathway for correction without adding components as the device is typically milled or printed as a single unit to avoid errors from luting titanium abutments [81, 82]. Therefore, it is best regarded as an adjunct rather than proof of fit. Digital verification is thus dependent on the scanning accuracy and reliable best‐fit alignment. Most available reports are technical descriptions with few clinical end‐points, and they largely address full‐arch workflows. The same principles also apply to short edentulous spans, but digital impressions for three‐unit implant‐supported fixed partial dentures have demonstrated trueness values under 75 μm which are within clinical tolerances, suggesting that verification is often unnecessary [97].

A hybrid approach digitises a passive IVJ fitted with scan analogues or an IVC with scanbodies and aligns the verified dataset to the IOS for comparison [56, 57, 58]. Agreement supports proceeding with fabrication, or the IOS data set can be merged with the laboratory scanned IVC data used for the relative implant positions. The clinical performance is dependent on the passivity of the physical index, the accuracy of the index scan, and the best‐fit alignment. Although they add additional laboratory steps and clinical time, they are useful in detecting and correcting discrepancies prior to the fabrication of the definitive prosthesis. For digital workflows that utilise titanium bases cemented into the framework, the IVC also forms an integral part of the workflow for the cementation process as an alternative to intra‐oral cementation which can be challenging with the risk of cement extrusion which needs to be meticulously cleaned to prevent peri‐implant diseases.

As an alternative to conventional impressions or IOS, photogrammetry is a digital method to record the relative three‐dimensional implant coordinates within an arch (Figure 6b). Under in vitro and in vivo conditions, photogrammetry shows higher trueness and precision than intra‐oral scanning or conventional impressions with verification procedures, which would indicate that conventional verification procedures would be counterproductive [20, 21, 22, 23, 24]. Photogrammetry scanners are able to perform self‐verification of precision by instantaneously assessing repeated measurements against each other; however, accurate seating and orientation of the scanning flags, device calibration, and patient and clinician‐related factors may still influence the outcome [64]. Emerging systems with assistive scanning devices (Figure 6c), horizontal scan bodies (Figure 6d), and AI‐based recognition report similar improvements in trueness and precision compared with conventional impressions and IOS with vertical scan bodies alone (Figure 6) [25, 26, 27, 28, 71, 74, 80]. As digital technology evolves and accuracy surpasses that of analogue techniques, it is difficult to justify routine verification with a physical index that is susceptible to polymerisation shrinkage and handling distortion as it may instead reintroduce errors.

Although several studies have reported high overall accuracy of IOS, there is a higher risk of extreme deviations in accuracy related to patient‐specific factors which may not be apparent at the time of scanning. Hence, for IOS, it is generally recommended that IOSs for edentulous patients should be verified with a hybrid approach or replaced with alternatives such as photogrammetry or horizontal scan bodies to minimise clinical misfit [9, 19, 20, 23, 95, 98].

The present narrative review notes several limitations, including the lack of evidence grading, an over‐representation of technical reports and in vitro studies, scarce clinical end‐points, and inconsistent definitions of passive fit or clinical thresholds. Together with the methodological heterogeneity of the included studies, these factors constrain the strength and generalisability of any clinical recommendations.

Against this backdrop, routine use of verification strategies—particularly verification jigs (IVJs) that rely on materials with stability limitations and are sensitive to operator technique, and that primarily detect gross misfit—warrants critical appraisal. The clinical utility of any verification step should be justified by the expected prevalence and magnitude of clinically relevant misfit, defined clinical thresholds and cost‐ and time‐effectiveness. At present, in vivo trueness data for IVJs are lacking, sectioned‐and‐re‐luted IVJ workflows have limited clinical evidence, and comparative studies do not consistently show trueness gains over well‐executed impressions. In many cases, verification procedures generate a second dataset without clear decision thresholds—functionally similar to taking two impressions—while adding cost and complexity. The use of verified casts as a reference for trueness in research evaluating the accuracy of digital scans or conventional impressions is commonly reported in the literature. However, given the errors and inaccuracy incorporated into the fabrication of verification devices, the trueness of the reference can be called into question and the results need to be interpreted with caution.

Future research should clearly define clinically meaningful misfit thresholds and whether verification procedures are necessary to improve clinical outcomes. Verification strategies should also be standardised, ideally with the development of objective clinical outcome measures for verification procedures. Until robust data link specific misfit thresholds and verification protocols to long‐term outcomes, clinicians should choose the analogue or digital pathway that minimises misfit while maintaining an efficient workflow, and reserve verification for scenarios with elevated risk of misfit or when predefined, clinically relevant thresholds are exceeded.

### 4.3 Clinical Recommendations

Practical guidance to minimise misfit across analogue and digital workflows is summarised below and is based on the evidence synthesised from current literature.

- *Conventional Impressions*: The use of IVJs for long‐span or full‐arch implant‐supported fixed dental prostheses is scientifically documented to reduce the risk of prosthesis misfit.
- *Digital IOSs with vertical scan bodies*: Although accuracies may approach conventional impressions with some reporting high variability, a modified verification protocol that fits into the digital workflow is recommended to reduce the risk of prosthesis misfit. Further research is required to define an optimal digital verification pathway.
- *Digital IOSs with horizontal scan bodies and AI‐based recognition*: Early clinical and in vitro studies show improved trueness and precision. However, the data is too scarce to recommend for or against the routine use of any specific verification pathway.
- *Photogrammetry*: Clinical and in vitro studies on photogrammetry demonstrate trueness and precision which surpass conventional verification pathways, negating the need for verification.

## 5 Conclusion

Within the limitations of this narrative review and the included studies, the use of IVJs for conventional impressions is well documented in the literature to reduce the risk of prosthesis misfit. However, the verification techniques reported are inconsistent and rely on subjective evaluations. The accuracy of intra‐oral scanning of extended edentulous spans with vertical scan bodies is subject to variability; thus, the modified use of verification protocols specific to digital workflows may be beneficial. Photogrammetry and intra‐oral scanning with horizontal scan bodies and AI‐based recognition demonstrate superior trueness and precision which negate the use of conventional IVJs. However, clinical judgement and meticulous assessment are still required to ensure passive fit.

## Author Contributions

**Adam Hamilton:** conceptualisation, visualisation, methodology, data curation, writing – review and editing, supervision. **Robert Nedelcu:** reviewing and editing, supervision. **Aaron Wai Harng Wong:** methodology, data curation, writing – original draft.

## Funding

The authors have nothing to report.

## Conflicts of Interest

The authors declare no conflicts of interest.

## Data Availability

The data that support the findings of this study are available from the corresponding author upon reasonable request.
